# Risk of COVID-19 in Children throughout the Pandemic and the Role of Vaccination: A Narrative Review

**DOI:** 10.3390/vaccines12090989

**Published:** 2024-08-29

**Authors:** David J. Weber, Kanecia O. Zimmerman, Sara Y. Tartof, John M. McLaughlin, Shanti Pather

**Affiliations:** 1 Division of Infectious Diseases, UNC School of Medicine, University of North Carolina, Chapel Hill, NC 27599, USA; 2 Duke Department of Pediatrics, Duke University School of Medicine, Durham, NC 27710, USA; 3 Department of Research & Evaluation, Kaiser Permanente Southern California, Pasadena, CA 91107, USA; 4 Pfizer Vaccines, 500 Arcola Rd, Collegeville, PA 19426, USA; 5 BioNTech SE, An der Goldgrube 12, 55131 Mainz, Germany

**Keywords:** SARS-CoV-2, COVID-19 vaccines, children, epidemiology, pediatric

## Abstract

At the beginning of the coronavirus disease 2019 (COVID-19) pandemic, persons ≥65 years of age and healthcare personnel represented the most vulnerable groups with respect to risk of infection, severe illness, and death. However, as the pandemic progressed, there was an increasingly detrimental effect on young children and adolescents. Severe disease and hospitalization increased over time in pediatric populations, and containment measures created substantial psychosocial, educational, and economic challenges for young people. Vaccination of children against COVID-19 has been shown to reduce severe acute respiratory syndrome coronavirus 2 (SARS-CoV-2) infections and severe outcomes in pediatric populations and may also help to prevent the spread of variants of concern and improve community immunity. This review discusses the burden of COVID-19 on children throughout the pandemic, the role of children in disease transmission, and the impact of COVID-19 vaccination.

## 1. Introduction

Children are an important population to consider in the global effort to protect against coronavirus disease 2019 (COVID-19). While infections and severe outcomes disproportionately affected older adult age groups early in the pandemic, children experienced potentially long-term detrimental consequences because of socioeconomic factors, such as disrupted school and social activities, and health factors, such as long COVID [1]. With the emergence of new Omicron sub-lineages, such as XBB [2] and the more recent JN.1 variant [3] and its descendants (e.g., KP.2 and KP.3) [4,5], clinical manifestations and transmission patterns of severe acute respiratory syndrome coronavirus 2 (SARS-CoV-2) have continued to evolve [6]. COVID-19 exacerbates the existing burden of pediatric hospitalization related to other respiratory viruses, such as influenza and respiratory syncytial virus (RSV) [7,8]. Despite the risk of COVID-19 in children, the spread of misinformation during the pandemic and fears related to adverse effects of the vaccine have contributed to a surge in vaccine hesitancy among the public, including in parents deciding whether to vaccinate their children [9,10,11].

SARS-CoV-2 variants of interest (VOIs) are defined as variants with genetic changes that are known or predicted to affect virus characteristics and that have a growth advantage relative to other circulating variants. VOIs that are associated with a detrimental change in disease severity or epidemiology, or a decrease in vaccine effectiveness, are known as variants of concern (VOCs) [12]. New VOIs and VOCs with increasing phylogenetic distance from the ancestral virus could enable sub-lineages to possess enhanced immune escape or the ability to evade neutralization by the immune system [6,13]. At the time of writing, the World Health Organization (WHO) declared that COVID-19 is no longer a public health emergency of international concern and provided recommendations for long-term disease management [14]. However, the WHO still tracks VOIs as the virus continually evolves [15]. XBB.1.5 and XBB.1.16 [16] have been replaced by JN.1 (an offspring of BA.2.86) and its descendants (e.g., KP.2 and KP.3) as the dominant variants globally [5,17,18]. The BA.2.86 variant has more than 30 mutations in the spike protein compared with the XBB sub-lineage, and the JN.1 offspring has an additional spike protein mutation; these alterations have demonstrated substantial immune evasive abilities [3,19]. Initial studies suggest that the KP.2 descendant of JN.1 has higher viral fitness and an increased immune escape ability compared to JN.1 [4].

This review provides an overview of the impact of COVID-19 on children and describes the evolution of risk that children have faced throughout the pandemic. Vaccination is emphasized as an important mechanism for protecting children from COVID-19. Barriers to vaccine acceptance and the reluctance of parents to vaccinate their children are also discussed.

## 2. Methods

References for this review were identified through searches of PubMed and the pre-print servers medRxiv and bioRxiv for English language articles. Additional sources of information included healthcare agency websites, ClinicalTrials.gov, and pharmaceutical company press releases. Additional publications were included on a case-by-case basis following the initial literature search and up to 15 March 2024, owing to the rapidly evolving nature of this topic. Some of the data reviewed here had not undergone peer review at the time of writing. The keywords used for the literature search were as follows: SARS-CoV-2, COVID-19 vaccines, epidemiology, pediatric, vaccination, vaccine, variant adapted, boosters, vaccine hesitancy, childhood vaccines, infection, transmission, symptoms, susceptibility, seroprevalence, age, adverse events, surveillance, vaccine safety, myocarditis, myopericarditis, multisystem inflammatory syndrome in children, MIS-C, MISC, MIS-A, MISA, pediatric inflammatory multisystem syndrome, PIMS-TS, long COVID, mental, adolescent, children, child, young adult, young adults, infants, school, and schools.

## 3. Burden of Disease in Children

Since the spread of Omicron B.1.1.529, which is known to have a higher transmissibility than the ancestral virus and Delta variant (B.1.617.2), several sub-lineages and descendent lineages with the potential for increased transmissibility, immune escape, and risk of re-infection have emerged. Multiple countries reported co-circulation of Omicron sub-lineages BA.2.12.1, BA.4, and BA.5, with the latter two variants having greater immune escape and displacing prior variants. Immune escape was also concerning with the XBB, XBB.1, XBB.1.5, and XBB.1.16 sub-lineages [20,21,22], and childhood cohorts exhibited higher rates of infection with XBB.1 and XBB.1.5 than other age groups [23,24]. JN.1, an offshoot of BA.2.86, was first detected in August 2023 and quickly became the dominant variant globally, with over 30 spike protein mutations that further enhance immune escape compared to the XBB lineage [3,19,25]. As of the time of writing, BA.2.86 and JN.1 are considered VOIs, and several circulating descendants of JN.1, including KP.2 and KP.3, are variants under monitoring [15].

Omicron sub-lineages have been associated with less severe clinical outcomes but a higher risk of transmission and greater immune escape from natural and vaccine-induced immunity than prior variants [20,21,26]. Data suggest variation in the rate of severe disease associated with Omicron sub-lineages, with infants and children <2 years of age bearing an increased proportion of severe illness [6,27]. Omicron BA.1 infection in both vaccinated and unvaccinated children resulted in lower antibody titers and function compared with the ancestral virus or Delta variant infection [28]. Higher antibody titers to Omicron infection elicited in vaccinated children compared with unvaccinated children support the role of vaccination in children in regard to inducing greater protective immunity [28]. Based on the absence of circulating original SARS-CoV-2 and the high prevalence of XBB lineages in 2023, the WHO Technical Advisory Group on COVID-19 Vaccine Composition (TAG-CO-VAC), the European Medicines Agency (EMA), and the United States Food and Drug Administration (FDA) recommended the use of monovalent, XBB-adapted vaccines for the 2023–2024 season, with a preference for the XBB.1.5 component [29,30,31]. The FDA approved two monovalent XBB.1.5 variant-adapted mRNA vaccines (BioNTech-Pfizer’s Comirnaty [BNT162b2] and Moderna’s Spikevax [mRNA-1273]) on 11 September 2023 for individuals ≥12 years of age, with emergency use authorization for children 6 months to 11 years of age [32]. The United States Centers for Disease Control and Prevention (CDC) Advisory Committee on Immunization Practices (ACIP) recommended all people aged ≥6 months receive the monovalent XBB.1.5 variant-adapted vaccine in the 2023–2024 season to protect against circulating variants [33]. In February 2024, the ACIP also recommended that adults ≥65 years of age receive an additional dose of monovalent XBB.1.5 variant-adapted vaccine due to increased risk for COVID-19-related severe illness [34]. In April 2024, the WHO TAG-CO-VAC advised that future COVID-19 vaccinations use a monovalent JN.1 lineage antigen [29]. The United States Vaccines and Related Biological Products Advisory Committee (VRBPAC) subsequently recommended a monovalent JN.1-lineage vaccine composition for the 2024–2025 formula of COVID-19 vaccines [35]. The FDA further determined that the preferred JN.1-lineage strain to include in vaccine targeting is KP.2, if feasible [36].

Data are emerging on the effect of monovalent XBB.1.5 variant-adapted vaccination or infection on antibody titers against XBB-lineage and newer variants, though information on the pediatric population is limited. At the time of writing, phase 3 trials of the XBB.1.5-adapted BNT162b2 mRNA vaccine in children and infants (NCT05543616) are underway [37]. Interim reports of phase 2/3 studies in the United States (NCT04927065 and NCT05997290) have demonstrated that adults who received a monovalent XBB-adapted mRNA vaccine developed robust neutralizing titers to XBB.1.5 [37,38], and no new safety signals or serious adverse events were reported in children 12–17 years of age [37]. Monovalent XBB.1.5 vaccination also elicited cross neutralization to other variants, including EG.5.1, BA.2.86, and JN.1 [37,38]. Multiple studies show that XBB.1.5 infection or monovalent vaccination induced neutralizing antibodies to JN.1 in adults; however, these antibodies were at relatively lower titers, which is consistent with the enhanced immune escape of circulating variants like JN.1 [39,40,41,42,43]. A study of previously vaccinated people who experienced a breakthrough infection during the BA.5-wave in China demonstrated that children and adolescents (<12 years and 12–18 years of age, respectively) had higher levels of neutralizing antibodies against JN.1 than adults (18–59 years of age) or seniors (>60 years of age) [43]. In adults and seniors, neutralizing titers to JN.1 increased significantly following re-infection during the XBB/EG.5 wave; re-infection was not studied in children, but these data suggest that exposure to the XBB.1.5 antigen can confer some cross-protection against circulating variants like JN.1. The WHO TAG-CO-VAC notes that preclinical data in mice and a single immunogenicity study in humans suggest that monovalent JN.1-containing vaccine candidates may elicit higher neutralizing antibodies to JN.1 and related variants (e.g., KP.2) than currently approved vaccines [29].

### 3.1. Prevalence

By to 1 January 2023, more than 75 million COVID-19 cases had been reported in children and adolescents <20 years of age, constituting 21% of 367 million infections in 105 countries [44]. Non-specific symptoms, such as cough and fever, which are the most common COVID-19-related symptoms in this population, are easily confused with other childhood respiratory illnesses (e.g., RSV and influenza) and may have led to limited testing of children for COVID-19 and under-reporting of infections. Reduced availability of COVID-19 tests may have also contributed to under-reporting, particularly during the early stages of the pandemic, when the availability of rapid detection tests was limited, and in certain countries throughout the course of the pandemic [45,46]. The true prevalence of prior SARS-CoV-2 infection in children is likely underestimated [47]. Serosurvey data from a sample of individuals ≤18 years of age extrapolated to the wider youth population in Mississippi has suggested the potential for infection with SARS-CoV-2 among 113,842 children. Within the same timeframe, fewer than 9000 confirmed and probable COVID-19 cases were reported [48].

### 3.2. Hospitalizations

Although the course of COVID-19 is usually milder or, more often, asymptomatic in children when compared with adults [49], increasing infection rates in this population have also been accompanied by an increase in hospitalizations [50]. The number of deaths caused by the disease in the first 5 months of the pandemic was higher than the annual deaths observed for several other pathogens prior to the introduction of effective pediatric vaccines (e.g., varicella and rotavirus) [51]. Data on hospitalization patterns in the presence of the highly transmissible JN.1 sub-lineage are emerging. Between 14 November 2023 and 3 March 2024, the UK Health Security Agency reported the lowest COVID-19-associated hospitalization risk in children 6–17 years of age (0.025%) and the highest risk in adults 75 years and older (3.3%) [52]. XBB-descendent lineages were the most prevalent at the beginning of this time period but were replaced by JN.1 in late 2023 [53,54]. In the United States, rates of COVID-19-associated hospitalization for the week ending 6 January 2024 peaked at 4.5 per 100,000 in those 0–4 years of age and 0.5 per 100,000 in those 5–17 years of age [55]. JN.1 and HV.1 were the dominant variants at the time of data collection [56]. In Israel, during the month between 11 February 2023 and 13 March 2024, weekly average hospitalizations among children were highest in those 0–4 years of age (0.5–1.4 per 100,000 persons) and lowest in those 12–15 years of age (0 reported per 100,000 persons) [57]. JN.1 was the most common sub-lineage when data were collected [58].

### 3.3. Clinical Manifestations

Similar to adults, a broad range of clinical manifestations of COVID-19 has been observed in children and adolescents 0–18 years of age [59]. Symptoms may differ depending on the variant; for example, there have been reports that cases of croup in children increased steeply with the incidence of Omicron BA.1 due to more upper respiratory tract infections with Omicron versus other variants [60,61]. Unlike previous variants, the Omicron BA.1 variant may present with febrile seizures in older children [62,63]; data on the occurrence of febrile seizures with Omicron sub-variants are currently lacking. Critical cases occurred in approximately 1% of children admitted to hospitals with diagnoses of acute respiratory distress syndrome, respiratory failure, or shock [59]. Omicron BA.1 and subvariants have been associated with increased rates of respiratory-related hospitalizations, especially in children <5 years of age and adolescents, compared with the Delta variant [64,65]. The XBB.1.16 variant has been linked with an increase in cases of non-purulent conjunctivitis [66]. Data on any symptoms that may be specific to JN.1 and KP.2 are not yet available. The WHO initial and follow-up assessments of JN.1 have not suggested any additional public health risks compared with other Omicron variants [3,67], and studies in adults have not revealed any additional risk of severe outcomes with JN.1 compared with XBB-descendant lineages [68,69].

Risk of severe outcomes is higher in children with underlying medical conditions than in healthy children [70]. In addition to severe respiratory symptoms during acute disease, children are also vulnerable to developing a hyperinflammatory syndrome (known as pediatric inflammatory multisystem syndrome, temporally associated with SARS-CoV-2 [PIMS-TS] in Europe and multisystem inflammatory syndrome in children [MIS-C] in the United States), which is an uncommon but severe complication of COVID-19, with as many as 70% of patients requiring care in an intensive care unit (ICU) [71]. The CDC have tracked reports of MIS-C since May 2020, and more than 9655 cases have been reported in the United States alone [72]. Importantly, the pathophysiology of MIS-C remains poorly understood, as do predictors of this severe illness. In a case series, >70% of affected children and adolescents were previously healthy [73]. This underscores the importance of immunization against SARS-CoV-2 in this population. Studies have shown that two vaccine doses reduce the risk of MIS-C in children 5–18 years of age [74,75]. In children >5 years of age with a history of MIS-C, vaccination against SARS-CoV-2 was not associated with a change in the safety profile [76]. The CDC recommends that people who are pregnant receive a COVID-19 vaccine due to evidence that maternal neutralizing antibodies can help protect infants from infection and hospitalization in the first 6 months of life [77,78,79,80].

### 3.4. Long COVID

Children, similar to adults, may develop long-term COVID-19 symptoms 4–12 weeks following acute symptomatic infection [81,82] and may retrospectively be referred to as ‘long COVID’ or post-acute sequalae. Long COVID may occur after infection with SARS-CoV-2 subvariants, including Omicron and Delta [83]. The increased risk of re-infection with Omicron subvariants, compared with re-infection with prior variants in children, may lead to longer-term health and learning implications related to symptomology associated with ‘long COVID’ or post-acute COVID-19 sequelae [84]. A systematic review and meta-analysis identified, with moderate certainty, the following several factors associated with an increased risk of long COVID in children: older age, allergic rhinitis, obesity, previous respiratory diseases, hospitalization, and severe and/or symptomatic acute COVID-19 [85]. Increased risk may also be associated with female sex, asthma, comorbidities, and heart diseases [85]. On the other hand, people who are Asian or Black may have a lower risk of long COVID [85].

In a survey of parents of children showing persistent symptoms after SARS-CoV-2 infection, the main symptoms were fatigue, weakness, headaches, abdominal pain, rash, and muscle and joint pain. These long-term symptoms lasted for more than 8 months on average. In addition, neuropsychiatric symptoms, such as difficulty concentrating, remembering, or processing information (also called ‘brain fog’), were reported [1]. A study from Russia showed that 24% of children admitted to hospital due to COVID-19 experienced persistent symptoms, such as fatigue, sleep disturbance, or sensory problems, after hospital discharge [86]. Persistent pulmonary damage has also been reported [87]. One study of 184 children in Israel reported that a mild obstructive pattern was the most common pulmonary impairment in children with persistent COVID-19 symptoms, affecting 11% of children even though most did not have asthma prior to infection [88]. The obstruction pattern was resolved within 1 to 6 months and, unlike adults, children did not have diffusion limitations. Most of the children with a resolved obstructive pattern were treated with inhaled corticosteroids, suggesting that it may be helpful to consider inhaler therapy for children with pulmonary function test abnormalities following COVID-19 [88]. A systematic review and meta-analysis that included studies from European and non-European countries estimated the prevalence of long COVID in children and adolescents to be 25% of those diagnosed with COVID-19 based on the presence of one or more symptoms for >4 weeks after infection with SARS-CoV-2. Among a variety of symptoms (>40) that may fluctuate in frequency and severity between patients, mood symptoms, fatigue, sleep disorders, headache, and respiratory symptoms were the most commonly characterized in long COVID [89].

## 4. Severity of COVID-19 Versus Influenza—A Comparison

In the United States, COVID-19 was the leading cause of death among infectious and respiratory diseases in children in 2021–2022 [90]. COVID-19 is often compared with influenza infections, as both are contagious respiratory illnesses and present with similar clinical features. However, studies have indicated that the health threat of COVID-19 in the pediatric population was greater than, or comparable with, that of influenza. One retrospective cohort study in 2020 showed that respiratory pathogenicity and mortality rates were higher for children and adolescents hospitalized with COVID-19 versus influenza [91]. In two population-based surveillance systems in the United States, it was shown that annual pediatric hospitalization rates and numbers of patients experiencing severe outcomes were equivalent to, or higher for, COVID-19 compared with influenza in seasons prior to the COVID-19 pandemic [7]. In 2022, mortality associated with COVID-19 was much higher than with influenza despite substantial decreases in COVID-19 death rates compared with the peaks seen in 2021 and 2020 [92].

Compared with previous years and during the COVID-19 pandemic, winter 2022–2023 was characterized by higher rates of seasonal influenza and morbidity, with a peak of nearly 17 hospitalizations per 100,000 individuals in England compared with pre-pandemic peaks of between approximately 6–8 hospitalizations per 100,000 individuals [93]. Several peaks in hospitalization rates for COVID-19 in England were observed throughout 2022–2023, with the highest at >20 hospitalizations per 100,000 individuals and another at approximately 12 per 100,000 individuals at the same time as the influenza peak [93]. Influenza hospitalizations returned to pre-COVID-19 pandemic levels in the 2023–2024 season. Though influenza hospitalization rates were highest among adults aged 75 and older, children 0–4 years of age were still greatly affected and hospitalized at a peak rate of more than 15 per 100,000 in the UK [94]. During the UK 2023–2024 season, the rates of RSV hospitalization were at the highest levels seen since the COVID-19 pandemic started, with hospitalization of children 0–4 years of age peaking near 40 per 100,000 [94].

Co-infection with SARS-CoV-2 and other respiratory pathogens, including influenza and RSV, may occur [95,96,97]. Among primarily hospitalized patients, co-infection with any pathogen is estimated to occur in almost 20% of those with SARS-CoV-2, and up to 41% of patients in ICUs experience superinfection (subsequent infection with other pathogens during care for SARS-CoV-2 infection) [98]. Viral co-infection was the most common, being present in 10% of patients with SARS-CoV-2, and influenza A was the most frequently identified viral co-infection [98]. Consecutive infections of influenza A followed by SARS-CoV-2 have also been reported [99]. Children may experience a higher rate of co-infection with community-acquired respiratory viruses and SARS-CoV-2 than adults [100]. Individuals with co-infection may also experience greater odds of death and increased likelihood of experiencing breathing difficulties [100]. Increased rates of influenza may increase the risk of co-infection with SARS-CoV-2 in the context of increased social contact and returning to school. Furthermore, because pediatric COVID-19 may present with non-specific symptoms, co-infection with influenza has been associated with misdiagnosis or underdiagnosis of COVID-19 in children with co-infection [101,102]. Influenza and RSV burden across age groups shows a clear seasonal pattern and SARS-CoV-2 spreads throughout the year with seasonal peaks in activity [93,94]; thus, co-infection with respiratory infections may become a seasonal paradigm. The seasonality of respiratory infections and the risk of co-infection in patients with SARS-CoV-2 may suggest a role for annual vaccination in pediatric populations.

The United States ACIP recommends that all individuals ≥6 months of age without contraindications should receive an annual influenza vaccine [103]. Regulatory bodies may be moving to COVID-19 vaccination approaches that resemble those established for influenza. On 26 January 2023, the United States VRBPAC voted to simplify COVID-19 vaccination recommendations for all age groups, including for children [104]. The VRBPAC also discussed the role of annual or biannual updated vaccination against SARS-CoV-2, with the potential for an additional dose (or doses) for young children and certain high-risk groups, including immunocompromised individuals. The 2023 WHO Strategic Advisory Group of Experts on Immunization (SAGE) roadmap for prioritization of COVID-19 vaccines outlined three priority use groups, primarily based on risk of severe disease and death. The low-priority group included healthy children and adolescents. Countries considering vaccination of children are urged to base decisions on contextual factors, such as disease burden and cost-effectiveness [105], while not compromising the routine vaccines that are crucial for the health and well-being of this age group [105]. The WHO subsequently published recommendations on transitioning from the emergency phase of COVID-19 to sustainable, comprehensive management for 2023 to 2025, which partly relies on preventing severe disease and death via vaccination [106]. In April 2023, the United States FDA amended the Emergency Use Authorizations of the BNT162b2 (COVID-19 vaccine manufactured by Pfizer/BioNTech) and mRNA-1273 (mRNA COVID-19 vaccine developed by Moderna and the Vaccine Research Center at the National Institute of Allergy and Infectious Diseases [NIAID] within the United States National Institutes of Health [NIH]) in order to authorize the bivalent Original/Omicron BA.4–BA.5 bivalent vaccines to be used for all doses in individuals ≥6 months of age; the authorization for the original vaccines was removed. The WHO TAG-CO-VAC recommended that vaccines for fall/winter 2023/2024 should contain an XBB descendent lineage [29]. The TAG-CO-VAC also advised moving away from the inclusion of the ancestral virus in the vaccines, as this virus is no longer circulating in human populations. The EMA and European Centre for Disease Prevention and Control (ECDC) stated that monovalent XBB-based vaccines could be administered as a primary series (two or three doses, depending on specific vaccine) in children <5 years of age and as a single dose in individuals >5 years of age when pediatric vaccination is recommended by national guidelines [31]. The FDA and EMA approved the updated monovalent XBB.1.5 variant-adapted vaccines for the 2023–2024 season and, at the time of writing, monovalent vaccines are the only ones approved for use in the United States [32,107]. The 2024–2025 COVID-19 vaccines will contain the JN.1 lineage (specifically the KP.2 strain, if feasible) based on recommendations from the WHO TAG-CO-VAC and United States VRBPAC [29,35,36].

## 5. Indirect Impact of COVID-19 on Children

During the pandemic, remote education, physical distancing, and ‘self-isolation’ led to increasing social isolation for children, with negative consequences on both physical and mental health [108] as well as on school performance [109]. The United Nations Children’s Fund (UNICEF) reported that daily exercise in children decreased and screen time increased, which were catalysts for weight gain and sleep disturbances [110]. In addition, stress and anxiety were increased in children and adolescents, not only reflecting the fear of infection but also uncertainty regarding education and development [110]. Both lack of activity and stress were shown to lead to increases in anger, irritability, and disruptive behavior in children, particularly those with attention deficit hyperactivity disorder (ADHD) or autism. Adolescents also showed moderate increases in depressive symptoms [110]. Alcohol and drug abuse in this age group also increased, likely as a coping mechanism [110]. Within households, cases of domestic violence also increased, and many families faced financial distress [111]. Food insecurity among households with children was exacerbated, often related to unemployment or lack of access to low-cost or free food due to school closures [112]. Health services were utilized less frequently, with negative consequences, including decreases in vaccination against other infectious diseases [111]. Indeed, the effects of the pandemic stretched far beyond the risk of acute infection. The reopening of schools and workplaces was anticipated to mitigate some of these other consequences of the COVID-19 pandemic; however, the data regarding this have been inconclusive. After a return to school among children 7–17 years of age in Portugal, signs of depression were reported in 15% of children, an increase in the pre-pandemic rate of 11% [113]. Nearly 20% of students 16–25 years of age returning to college in China reported depression, anxiety, acute stress, or insomnia alongside their return to school, rates that were similar to those experienced during the COVID-19 pandemic outbreak phase [114]. Though COVID-19 is now considered endemic, the long-term impact of the pandemic on children may persist.

## 6. Transmission

From December 2023 to March 2024, adults and children were affected by rapid transmission of JN.1 and its parent lineage BA.2.86 [115,116]. BA.4.6 and XBB.1.5 were dominant in the prior year prior [23,24]. Omicron has spread more rapidly than previous variants, with a doubling time of 2.5 days [117]. The doubling time for XBB.1.5 is estimated at 9 days; however, the XBB lineage exhibits a high growth advantage, most likely driven by immune escape properties and changes to the viral spike protein [118]. XBB.1.16 may have a higher reproductive number than XBB.1.15 [22]. JN.1 is highly transmissible, likely due to multiple spike protein mutations that drive enhanced immune escape [3]. The ECDC notes that the success of BA.2.86-descendant lineages is likely driven by immune escape in a population that has increasingly developed immunity to XBB-variants [119].

It has been demonstrated that children play a role in the increased household transmission of respiratory and gastrointestinal infections [120]. Unvaccinated children may also facilitate the transmission of SARS-CoV-2 given that at least 50% of pediatric cases are asymptomatic and, thus, the individuals unknowingly spread the virus [121]. COVID-19 vaccination may reduce disease transmission [122]. The duration of protection against SARS-CoV-2 variants, such as Omicron, is still being established and may be of limited duration [123]; however, lower rates of vaccination among children may lead to the spread of Omicron sub-lineages in schools [124]. Furthermore, the absence of symptoms in combination with high viral loads, potentially further enhanced by mixing patterns in high-contact settings such as indoor school environments, may not only make unvaccinated children one of the drivers for disease transmission but may also trigger the formation of even more transmissible variants [125]. A study in Japan found that young children 0–3 years of age experienced an 8–10-fold increase in COVID-19 transmission during the Omicron outbreak compared with earlier strains (B.1.1.284 and B.1.1.214) of SARS-CoV-2 [126]. Children 0–3 years of age were not required to wear masks in pre-schools and experienced higher rates of secondary infections than children 4–6 years of age who were required to wear masks at pre-school/school during the Delta wave. A similar risk of transmission was seen between the two age groups during the Omicron wave, highlighting the increased transmission efficiency of Omicron and the need for additional mechanisms beyond mask wearing, such as vaccines, to protect young children from Omicron and its evolving sub-lineages [126].

In some regions, schools contributed to increasing infection rates. For example, in England, the number of SARS-CoV-2 infections increased exponentially in children and adolescents 5–17 years of age when schools were reopened at the start of the autumn term [127]. In Austria, the 7-day incidence for children and adolescents 5–14 years of age increased to >2300 cases per 100,000 population in November 2021, calculated using population figures for each age group, the highest level of all age groups [128]. In the United States, it has been shown that secondary transmission can be reduced in schools with high adherence to mask wearing and vaccination [129]. One study showed that secondary transmission in the community was higher than that in schools with universal masking measures [130], and a meta-analysis of contact tracing studies in 112,622 subjects in schools revealed a limited viral transmission of 3% [131]. Studies that systematically tested children demonstrated infection rates similar to and, in some settings, higher than adults. Prior to the vaccination of individuals 12–17 years of age, school outbreaks occurred but were lower than other settings when preventative measures were in place [132]. Several studies have found evidence of differing transmission dynamics between younger versus older children, with higher transmission seen in older children, which may be, in part, due to participation in team sports [133]. This is in line with a study based on infection data from 637 households in Benin Brak, Israel, which showed that the transmission rates in those 15–19 years of age were comparable with those of adults at a time when schools were open [134]. Data describing household transmission from children to adults are limited and are dependent on the dominant variant at the time of data collection. In the early phase of the pandemic, only a limited number of household transmission clusters (3.8%) were reported involving children and adolescents (<18 years of age) as the index case [135]. A retrospective assessment of the transmission of SARS-CoV-2 acquired by children and adolescents 7–19 years of age at an overnight camp to household contacts showed that transmission occurred in 18% of households [136].

## 7. Vaccination in Children—Benefits and Recommendations

In certain countries, children receive a yearly influenza vaccination as a routine measure. This is not only because influenza can lead to severe illnesses, hospitalizations, and even deaths in very young children, but also because children are likely to transmit the virus to vulnerable adults [137]. Similarly, the potential for severe illness or transmission to adults is an important rationale for vaccinating children against SARS-CoV-2. Vaccination also protects children against severe cases of COVID-19 and MIS-C [138,139] and provides greater protection against re-infection than naturally acquired immunity, according to data from adults, because the antibody response from vaccination is higher than from natural infection and lasts longer [50]. Antibody titers are lower in individuals with mild or no symptoms, which is often the case for children. As such, vaccination may offer greater protection from re-infection than initial infection with SARS-CoV-2.

Reducing hospitalizations through vaccination also contributes to relieving the burden on overwhelmed healthcare systems. The COVID-19-Associated Hospitalization Surveillance Network (COVID-NET) conducted population-based surveillance and showed that, during periods in which Delta and Omicron were predominant, hospitalization rates were lower among fully vaccinated adolescents 12–17 years of age than among unvaccinated adolescents [140]. The risk of Omicron BA.1-associated hospitalization among children 5–11 years of age was reduced by two-thirds following BNT162b2 vaccination. Among adolescents 12–18 years of age, protection against Omicron-associated hospitalization was lower than against Delta-associated hospitalization following two doses of BNT162b2, but critical illness caused by either variant was prevented [141]. BNT162b2 vaccination, especially with two or three doses, provided protection against hospitalization and emergency department visits related to BA.2 infection in adults [142] and provided protection against emergency and urgent care visits in children 5–11 years of age [143]. As assessed from a real-world cohort analyzed during periods of BA.1, BA.2/BA2.12.1, and BA.4/BA.5 predominance and co-circulation, vaccine effectiveness against Omicron in children was 60% shortly after receipt of two doses of BNT162b2 and nearly 80% shortly after a booster dose [143]. In a study in Singapore featuring children previously infected with SARS-CoV-2, the effectiveness of two doses of the BNT162b2 vaccine against the XBB sub-lineage was 62.8% in those 5–11 years of age and 47.9% in those 12–17 years of age [144]. Data from the United States suggest that the emergence of XBB descendent lineages, including XBB.1.5, led to a decrease in COVID-19 mRNA vaccine effectiveness in children <11 years of age, highlighting the need for variant-adapted vaccines [145].

Furthermore, the vaccination of children may help protect the general population by reducing the spread of SARS-CoV-2 [121]. This is in line with other pathogens for which the vaccination of children has shown indirect benefits for the whole population (e.g., hepatitis A, rotavirus, rubella, and pneumococcal disease, including reducing pneumococcal disease in adults) [51]. Pediatric vaccination against SARS-CoV-2 may also help reduce school closures, which are known to interrupt learning, create gaps in childcare, affect the nutrition of children who rely on school meals, and affect families economically due to parents missing work during the pandemic [146]. A study of children <12 years of age in North Carolina in the United States from 29 October 2021 to 6 January 2023 found that BNT162b2 and mRNA-1273 primary vaccines and boosters were effective against Omicron infection and severe outcomes [147]. Vaccination with bivalent boosters was more effective than monovalent boosters [147]. A previous study of over 7000 children aged 6 months to 4 years in the United States found that receipt of at least two COVID-19 mRNA vaccine doses was 40% effective in preventing hospitalization and emergency department visits at a time when several Omicron sub-lineages were circulating [148]. Though this study was conducted prior to the development of the monovalent XBB.1.5 variant-adapted vaccines, the results suggest that COVID-19 vaccination can continue to protect children from severe outcomes and hospitalization.

Monovalent XBB.1.5 variant-adapted mRNA vaccines were approved for use in the autumn/winter 2023/2024 season in the Northern Hemisphere [32,149]. Though pediatric data are currently limited, recent studies have examined the effectiveness of the monovalent XBB.1.5 variant-adapted mRNA vaccine in preventing infections and hospitalization in adults. A study of over 6500 adults in the United States found that participants who received the monovalent XBB.1.5 mRNA vaccine had a 72% reduced risk of an inpatient or emergency department visit versus an outpatient visit when infected with an XBB-lineage variant; these odds were limited to 38% when infected with JN.1 [150]. Two other studies of Danish and French adults reported no differences in risk of hospitalization or severe outcomes between XBB variants and JN.1 [68,69]. How these findings apply to the pediatric population during JN.1 circulation remains to be seen. Nonetheless, prevention of co-infection or consecutive infection through the use of variant-adapted vaccines in children may improve outcomes in a COVID-19 endemic setting with high circulations of other respiratory viruses.

### 7.1. Approvals

Based on expanding data on the safety and effectiveness of COVID-19 vaccination in pediatric populations [151,152,153], original COVID-19 mRNA vaccines were approved by the FDA and EMA as a primary series in children ≥6 months of age [154,155]. Following the development of the bivalent mRNA original/BA.4-BA.5 vaccines, the FDA simplified the Emergency Use Authorizations for the mRNA vaccines to recommend the bivalent vaccines for all doses in individuals ≥6 months of age, including as a primary series in unvaccinated children 6 months to 5 years of age [107]. On 6 December 2022, the EMA Emergency Task Force issued a statement confirming that bivalent mRNA original/BA.4-BA.5 vaccines, which had been authorized as booster vaccinations, may also be used as a primary series, including for certain children [156]. More recently, the FDA and EMA have approved monovalent XBB.1.5-adapted vaccines; these are currently the only COVID-19 vaccines approved for use in the United States [32,149,157]. However, the WHO TAG-CO-VAC and United States VRBPAC recommended that 2024–2025 COVID-19 vaccine formulations use a monovalent JN.1 lineage antigen, with KP.2 being preferred, if feasible [29,35,36].

Omicron variant-adapted vaccines prolong the duration of protection and reduce disease transmission. Vaccine effectiveness with the original vaccines waned against symptomatic disease with Omicron BA.1 and BA.2 at 1-month post-booster, but vaccine effectiveness against hospitalization remained high, although protection beyond 6 months is unknown. Variant-adapted vaccines may increase vaccine effectiveness against symptomatic disease, provide greater protection against variants with immune escape mechanisms, and offer more durable protection [158]. During the BA.5 wave, older adults vaccinated with a bivalent BA.4/BA.5 booster following a primary vaccine course experienced lower rates of hospitalization and mortality than those protected with monovalent vaccines alone [159].

Although the overall severity of disease may have decreased following the emergence of Omicron sub-lineages [160], certain age groups may show relatively higher rates of infection with emerging variants. For example, the relative infection rate of XBB.1.5 was initially highest in children 10–19 years of age compared with other age categories, but the infection rates of BA.2.86 and JN.1 are currently similar across ages 10 to 49 [24,115,116]. Owing to the ongoing evolution of SARS-CoV-2 and the emergence of variants that were antigenically distant from prior vaccines, the WHO, EMA, and FDA recommended an update to vaccine composition for the autumn/winter 2023/2024 season in the Northern Hemisphere that incorporated an XBB component, with emphasis being placed on XBB.1.5 [29,31,161]. These vaccines are monovalent because the ancestral SARS-CoV-2 virus and closely related variants are no longer circulating in human populations [29]. The FDA approved the use of monovalent XBB.1.5 variant-adapted vaccines on 11 September 2023 for use in individuals >12 years of age and issued an emergency use authorization for children 6 months to 11 years of age [32]. The EMA also approved XBB.1.5 variant-adapted mRNA vaccines in August and September of 2023, and these are authorized in individuals 6 months and older [149]. An overview of currently approved vaccines is available in Table 1.

### 7.2. Vaccine Risks

mRNA COVID-19 vaccines have a good safety profile in children, including those <5 years and 5–11 years of age [167,168]. In children <5 years of age who received the BNT162b2 vaccine, the most frequent adverse events included local injection-site symptoms and were not different from those experienced with other types of routine childhood vaccination [167]. Most post-vaccination symptoms were of no or minimal severity and did not lead to children missing school or the need for childcare [167]. Among uncommon events, myocarditis and pericarditis were rarely experienced, and the event risk was not different between BNT162b2 and mRNA-1273 [169]. A systematic review and meta-analysis of myopericarditis in adolescents and young adults (12–20 years of age) after vaccination with an mRNA COVID-19 vaccine reported an overall incidence of approximately 0.3–5.0 cases per 100,000 vaccinated individuals, with events occurring primarily after the second vaccination in male adolescents [9]. As the risk of developing myocarditis after SARS-CoV-2 infection (11 events per 100,000 persons) is higher than the risk after COVID-19 mRNA vaccination, the benefits of mRNA COVID-19 vaccination may outweigh potential risks [9]. Furthermore, the safety profiles of bivalent mRNA COVID-19 vaccines administered as a booster were similar in individuals ≥12 years of age to those of monovalent boosters and were favorable compared with the potential health impacts associated with COVID-19 [170]. An interim report of a phase 2/3 study found no new safety signals and no severe adverse events from a monovalent XBB.1.5 variant-adapted vaccine in children 12 to 17 years of age [37].

### 7.3. Vaccine Implementation and Acceptance

Despite there being a lack of safety concerns regarding COVID-19 mRNA vaccination in children, vaccine acceptance among parents has been low [9]. As of 16 March 2024, the CDC reports that only 136% of children have received the updated 2023–2024 COVID-19 vaccine [171]. Some parents feel that COVID-19 vaccines are less important or effective for their children than routine childhood vaccines and cite inadequate safety information on the COVID-19 vaccine as a primary reason for hesitancy [172]. Rare cases of post-vaccination myocarditis, although mostly mild and transient [173], have likely further amplified parents’ skepticism about vaccinating children. Myocarditis, which occurs most frequently in young males [174], is an important concern, but the risk of myocarditis is substantially higher in unvaccinated individuals who become infected with SARS-CoV-2 than the risk of myocarditis seen after one or more doses of the BNT162b2 vaccine [173], which is an important point to communicate to hesitant parents.

The WHO has described vaccine hesitancy as one of the top 10 threats to global health [175]. The lack of vaccine acceptance has complex origins, including behavioral, cultural, political, and educational factors, as well as other drivers such as fear, misinformation, complacency, and convenience [176]. The erosion of public trust in healthcare systems during the COVID-19 pandemic resulted, in part, from surges in the spread of misinformation and has been detrimental to vaccine acceptance [177]. Though a global issue, there may be disparities in patterns of and motivations for vaccine hesitancy between high-income countries (HICs) versus low- and middle-income countries (LMICs). A study analyzing data from 44,260 individuals across 15 survey samples reported substantially greater willingness to receive a COVID-19 vaccine in LMICs compared with Russia and the United States (HICs) [178]. The correlation between economic status and vaccine hesitancy appears largely contextual and thus difficult to predict. In the case of COVID-19, opinions in HICs are predominantly related to vaccine safety concerns and may be driven by politicization of the specific pandemic and vaccine. Conversely, determinants of vaccine hesitancy in LMICs are more frequently related to cost, access, and awareness, which may rely more heavily on anecdotal evidence [11,178]. Some parents remain reluctant in regard to pediatric vaccination, and there is a tendency to question scientific consensus. A trend of decreasing vaccination rates, even for well-established vaccines approved in the pediatric population, has already been observed prior to the COVID-19 pandemic, as described by a study in the Netherlands [179]. The spread of misinformation and concerns regarding adverse events and long-term safety can also result in vaccine hesitancy.

Another concern may relate to previous SARS-CoV-2 infection and whether vaccination is still necessary. Many children may have already been infected with SARS-CoV-2 during the pandemic, particularly during the Omicron BA.1/BA.4/BA.5 and XBB.1.5/XBB.1.16 waves, owing to their highly transmissible nature. Nevertheless, it is still important to initiate widespread vaccination campaigns in this group, as risk remains in the current endemic setting due to the ongoing evolution of SARS-CoV-2 resulting in circulation of antigenically distant variants. Vaccination following infection has been shown to significantly increase serum neutralizing activity against Omicron [180]. In adult populations, regardless of SARS-CoV-2 infection history, higher neutralizing titers have been seen with bivalent BNT162b2 against Omicron variants descended from BA.5 and BA.2, including BA.2.75.2, BQ.1.1, and XBB.1, than seen after monovalent booster vaccination [181]. Furthermore, after a booster dose, those with prior SARS-CoV-2 infection had higher neutralizing titers than those without prior SARS-CoV-2 infection [181]. Additionally, the updated monovalent XBB.1.5 variant-adapted vaccine has also been shown to elicit a robust neutralizing antibody response in adults, with cross-neutralization for other variants such as BA.2.86 and JN.1 [37,38,39,40]. How these findings apply to children remains to be seen. Nonetheless, the continuing evolution of SARS-CoV-2 supports the use of variant-adapted vaccines. For example, immunocompromised adults who received the 2023–2024 COVID-19 vaccine experienced a lower risk of hospitalization than those who did not receive an updated vaccine [182]. Additionally, natural immunity to JN.1 following infection was estimated to decrease to nearly 50% after 6 to 9 months and further decreased to negligible levels of protection after one year [183]. These studies highlight the necessity of updated vaccines to protect both immune-competent and immunocompromised individuals from new variants. Vaccination remains important, even in individuals with prior SARS-CoV-2 infection. Waning effectiveness of both vaccination and prior infection over time further supports the need for booster doses of variant-adapted vaccines [184,185] to provide protection against circulating variants in pediatric populations prior to potential seasonal surges.

Vaccinated parents can show hesitancy towards vaccinating children aged 5–11 years due to their belief that COVID-19 presents a low risk to children [186]. A systematic review and meta-analysis of factors influencing parental willingness to vaccinate their children against COVID-19 determined that parents demonstrated lower rates of intention to vaccinate their children compared with that of the general population to self-vaccinate [187]. The authors speculated that the perception that COVID-19 presents a low risk to children may underpin these intentions [187]. A trial that randomized unvaccinated individuals to receive targeted messages about the benefits of COVID-19 vaccination tailored to their identity, including their role as a parent, resulted in no substantial effect on willingness to vaccinate themselves or their children [188].

Several strategies have been outlined to promote pediatric vaccination acceptance and uptake, applicable both for COVID-19 vaccines and in wider vaccination programs. These include vaccination incentives and mandates, use of presumptive language by healthcare providers when initiating vaccine discussions, vaccination reminders or scheduling, and implementation of on-site vaccination in schools [189]. Healthcare professionals often serve as the principle and most trusted source of information for parents and thus play a crucial role in tackling misinformation related to vaccines. By providing accurate information and engaging in open discussions with parents to address their concerns, healthcare professionals can help promote vaccine acceptance [178,190].

## 8. Conclusions and Perspectives

Children remain at risk of severe outcomes of COVID-19 in the current endemic setting, particularly when other respiratory pathogens are circulating, increasing the likelihood of co-infection [98]. Vaccination of children has been shown to be effective against SARS-CoV-2 infection and severe COVID-19 [140]. It is also likely to help prevent long-term consequences of COVID-19 in children. Previously established mitigation measures, such as social distancing and school closure, exacted a heavy toll on the psychosocial health of children and adolescents [108]; these non-pharmaceutical interventions, alongside mask wearing, have decreased in use. Thus, vaccination has become even more important in helping to protect younger populations from COVID-19. Omicron and currently circulating XBB- and BA-descendent sub-lineages such as JN.1 are more transmissible than prior variants, increasing risk of infection, and subsequently severe outcomes, in children and other populations. Though SARS-CoV-2 has transitioned into an endemic setting, the virus is expected to continue to evolve, resulting in the circulation of antigenically distant variants. Vaccination of high-risk populations, including children, as well as booster doses for those previously vaccinated with waning immunity, is necessary to help mitigate the ongoing risk. Large pediatric vaccination campaigns with variant-adapted vaccines may face challenges related to vaccine access, as demand has increased globally. In other parts of the world, parental concerns about vaccine reactogenicity, uncommon adverse events, or lack of long-term safety data may hinder vaccination progress. These concerns are further fueled by vaccine misinformation, which may be propagated by the media. Recent guidance from public health advisory bodies indicates that COVID-19 vaccination may adopt an annual pattern similar to influenza vaccination in the coming years. Vaccination implementation strategies, including accurate dissemination of scientific information, need to be carefully considered, weighing benefits versus risks, as COVID-19 vaccination will likely have far-reaching impacts on all vaccination programs worldwide.

## Figures and Tables

**Table 1:** Summary of approved vaccines by age group.

Vaccine Type	Vaccines	Approved Age Group [149,157]	Most Common Adverse Reactions
mRNA	Comirnaty (Pfizer and BioNTech) [162]	EU: ≥6 months old *	Local: injection site pain, swelling Systemic: tiredness, headache, muscle pain, joint pain, chills, fever, diarrhea
		US: ≥12 years old ^†^	
		UK: ≥6 months old	
	Spikevax (Moderna) [163]	EU: ≥6 months in EU *	Local: stiffness/pain/swelling/redness at injection site Systemic: swelling/tenderness in the underarm, decreased appetite, irritability/crying, headache, sleepiness, nausea, vomiting, muscle ache, joint aches, feeling very tired, chills, fever
		US: ≥12 years old ^†^	
		UK: ≥6 months old	
Protein subunit	Nuvaxovid (Novavax) [164]	EU: ≥12 years old	Local: tenderness or pain at injection site Systemic: headache, nausea/vomiting, muscle ache, joint pain, fatigue, generally feeling unwell
		US: ≥12 years old	
		UK: Not approved	
	Bimervax (HIPRA Human Health) [165]	EU: ≥16 years old	Local: pain at injection site Systemic: headache, fatigue, muscle pain
		US: Not approved	
		UK: ≥16 years old	
Vector	Jcovden (Janssen) [166]	EU: Not approved	Local: pain at injection site Systemic: headache, nausea, muscle ache, feeling very tired
		US: Not approved	
		UK: Not approved in children (≥18 years old)	

* Excluding original strain + Omicron BA.1 variant (adapted) vaccine, which is individuals ≥12 years old; ^†^ emergency use authorization of 2023–2024 formula for individuals ≥6 months to 11 years old. mRNA, messenger RNA; UK, United Kingdom; US, United States; EU, European Union.
